# What are essential elements of high-quality palliative care at home? An interview study among patients and relatives faced with advanced cancer

**DOI:** 10.1186/s12904-019-0485-7

**Published:** 2019-11-06

**Authors:** M. G. Oosterveld-Vlug, B. Custers, J. Hofstede, G. A. Donker, P. M. Rijken, J. C. Korevaar, A. L. Francke

**Affiliations:** 1 Nivel, Netherlands institute for health services research, P.O. Box 1568, 3500 BN Utrecht, The Netherlands; 2 Expertise Center Palliative Care VU University Medical Center, Amsterdam, The Netherlands; 3 Amsterdam UMC, Vrije Universiteit Amsterdam, Amsterdam Public Health research institute, Amsterdam, The Netherlands

**Keywords:** Palliative care, Primary health care, Home-care services, Cancer, Quality of care

## Abstract

**Background:**

In the Netherlands, general practitioners (GPs) and community nurses play a central role in the palliative care for home-dwelling patients with advanced cancer and their relatives. To optimize the palliative care provision at home, it is important to have insight in the elements that patients and relatives consider essential for high-quality palliative care, and whether these essentials are present in the actual care they receive.

**Methods:**

Qualitative semi-structured interviews were conducted with 13 patients with advanced cancer and 14 relatives. The participants discussed their experiences with the care and support they received from the GP and community nurses, and their views on met and unmet needs. Interview data were analysed according to the principles of thematic analysis.

**Results:**

Patients as well as relatives considered it important that their GP and community nursing staff are medically proficient, available, person-focused and proactive. Also, proper information transfer between care professionals and clear procedures when asking for certain resources or services were considered essential for good palliative care at home. Most interviewees indicated that these essential elements were generally present in the care they received. However, the requirements of ‘proper information transfer between professionals’ and ‘clear and rapid procedures’ were mentioned as more difficult to meet in actual practice. Patients and relatives also emphasized that an alert and assertive attitude on their own part was vital in ensuring they received the care they need. They expressed worries about other people who are less vigilant regarding the care they receive, or who have no family to support them in this.

**Conclusions:**

Medical proficiency, availability, a focus on the person, proper information transfer between professionals, clear procedures and proactivity on the part of GPs and community nursing staff are considered essential for good palliative care at home. Improvements are particularly warranted with regard to collaboration and information transfer between professionals, and current bureaucratic procedures. It is important for care professionals to ensure that the identified essential elements for high-quality palliative care at home are met, particularly for patients and relatives who are not so alert and assertive.

## Introduction

Persons with advanced cancer and their families are a key target group for palliative care. International research has established that most people with advanced cancer would like to remain at home among family until they die [1–3]. Many patients are indeed able to remain at home until the end, although there are cross-country differences. Ko et al. (2014) analysed the records in four countries of more than 2000 patients who died of cancer and found that home deaths were more common in the Netherlands (57.9%) than in Spain, Italy and Belgium (resp. 51.1, 45.5 and 33.2%) [4].

The fact that a relatively large proportion of patients with cancer in the Netherlands die at home instead of in hospitals or other inpatient settings, is related to the fact that this country has a generalist palliative care model [5]. This means that healthcare is characterized by a strong emphasis on primary care, where the general practitioner (GP) is the central professional in the medical care for community-dwelling patients, at all stages of life. GPs play a pivotal role in offering palliative care at home, often in cooperation with community nursing staff. In the event of complex problems, such as managing refractory symptoms, they have opportunities to consult specialist palliative care teams by telephone, which are available all over the country [6].

Because of the central roles of GPs and community nursing staff in delivering palliative care at home in the Netherlands, it is important to know how patients and families perceive the quality of this care, and whether it fulfils their needs. The perceived quality of palliative care is often measured by asking about satisfaction with care, generally resulting in high satisfaction with almost all aspects of care e.g. [7, 8]. However, asking about care satisfaction may lead to socially desirable responses, because care users are often grateful to and dependent on their care providers. Exploring what care users find important aspects of good-quality palliative care and relating this to their actual experiences may provide a more valid picture and may also provide insight into the care aspects where quality improvements are most needed. For this reason, Heins et al. (2018) performed a survey study using parts of the Palliative Care Consumer Quality Index [9] in which advanced cancer patients’ actual care experiences were compared to the ratings they gave for the importance of 13 items regarding information provision, respect for their autonomy and support when suffering from pain or other symptoms [10]. Seventy-two home-dwelling Dutch patients with advanced cancer participated, and almost all rated all 13 items as important. In general, the palliative care that patients received matched with what they considered as important. However, improvements could be made in the provision of support to patients suffering from fatigue and in the provision of information on the expected course of patients’ illness [10]. A limitation of this quantitative survey was, however, that respondents had limited opportunities to give in depth-information about their perspectives or to mention new topics that are also important to them.

Therefore, we took a subsample of the people who participated in this survey study, and performed an additional qualitative interview study with the following research questions:

- What do patients with advanced cancer and their family consider essential for good-quality palliative care as delivered by GPs and community nursing staff in the Netherlands?
- Does the actual palliative care provided match what these patients and their family consider essential for good-quality palliative care, and what contributes to this?

## Methods

### Design

The research questions were addressed in semi-structured qualitative interviews with patients with advanced cancer and with relatives of patients with advanced cancer.

### Sample and recruitment

The interviewees were recruited within the framework of the afore-mentioned quantitative survey study [10]. For this survey study, a large sample of GPs − selected from the national GP registry − were asked whether they were willing to recruit one or more of their advanced cancer patients with a limited life expectancy based on the ‘surprise question’: “Would you be surprised if this patient died in the next 12 months?“ [11] In this way, 72 patients and 63 relatives were recruited between June 2015 and January 2016 and participated in the survey study. Of them, 28 patients and 22 relatives stated in the survey that they would also be willing to participate in an additional interview. Because of rapid deterioration in health or the patient’s death, 13 patients and 14 relatives could finally be interviewed between September 2015 and May 2016.

### Setting

All interviewees received medical care from a GP, often combined with care from community nursing staff. In principle, all residents in the Netherlands are registered with a general practice. The GP is responsible for the medical care of home-dwelling patients and acts as a gatekeeper for hospital and specialist care. GPs offer out-of-hour services by GP cooperatives across the whole country and are thus available day and night, seven days a week [12].

Community nursing includes care at home that is related to a need for medical care. Both personal care and nursing care fall under community nursing. A bachelor-educated community nurse performs the formal needs assessment procedure with the patient and the family representative, but the actual care is often provided by nurses or professional carers trained to secondary vocational level. Together, the afore mentioned professionals form the community nursing team [13]. Both GPs and community nursing staff deliver psychosocial support as well. Both types of care are covered by the mandatory public health insurance in the Netherlands.

### Data collection

The interviews were partly conducted by one of the co-authors (JH, sometimes together with BC) and partly by trained interviewers. The interviews were guided by a topic list, including questions on patients’ and relatives’ experiences with the care and support they received from their GP and/or community nursing team and their views on met and unmet needs regarding the care they received. The interviews took place at the interviewees’ home, lasted 30–60 min and were audio-taped and transcribed verbatim.

### Data analysis

Data analysis started during data collection and was an ongoing process. The transcripts were coded with the aid of MAXQDA software. Following the principles of thematic analysis [14], transcripts were first read and re-read to become familiar with the data. Then codes were ascribed to meaningful text units, and codes were grouped together to search for themes among them. At some point, the evolving code list remained unchanged at each subsequent interview, indicating that data saturation has been achieved. The emerging themes were constantly compared with the content of the interview transcripts. This process revealed that multiple linkages between emerging themes existed. To identify overarching themes and to visualize their interrelatedness, we made use of a visual concept map. To ensure reliability of the coding procedure, about half of the interviews were coded independently by the second author and one of the other co-authors. This revealed a high degree of consensus between the different coders; any disagreements were solved by discussion with other members of the research team.

## Results

### Characteristics of participants

The patients who were interviewed were aged between 58 and 86. Nine of the 13 patients were male. The primary tumour sites were the lungs (*n* = 5), colon, liver, breasts, kidney, prostate, ovaries, lymph nodes and the neuro endocrine system (all *n* = 1). The interviewed relatives were aged between 40 and 79. The majority (11 of the 14 relatives) were female. Nine relatives were the partner of a patient with advanced cancer, four were daughters and one was a sister of a patient. The relatives were not necessarily related to one of the interviewed patients; but in nine cases both the patient and their relative (partner or daughter) were interviewed − separately from one another. More than half of the interviewees were highly educated (58% of the patients and 67% of the relatives).

### Essentials of good-quality palliative care at home

The interviews revealed six essential elements for good-quality palliative care provided at home (Table 1). In the following sections we explain how these essential elements were described by the interviewees. We also show that these essential elements were often realized in practice, but certainly not in all cases.

**Table 1:** Overview of the essential elements of good-quality palliative care at home

Themes	
1	Medical proficiency
2	Availability
3	A focus on the person - Showing personal interest - Taking patient and relatives seriously - Having a bond of trust
4	Proactivity - Making timely arrangements with other care providers - Informing patient and relatives in good time - Openly discussing end-of-life care
5	Proper collaboration and information transfer between professionals
6	Clear and rapid procedures

#### Theme 1: medical proficiency

Interviewees repeatedly mentioned the medical competence of their GP and community staff as an indicator to evaluate whether they were receiving good care. GPs who had already demonstrated their knowledge and skills earlier in the diagnosing process often created a feeling of trust with the patient and relatives for the remainder of the disease and care trajectory. In contrast, those who had experienced a doctors’ delay in the diagnosing phase expressed a lack of trust in their GP’s professional medical competencies in subsequent stages. Some participants mentioned that the GP had limited experience with their specific type of cancer type. With regard to the community nursing staff, most interviewees said that they did what they were expected to: they worked professionally, were hygienic and showed competence in, for instance, the prevention of pressure sores.

*“They are very respectful, and I also feel that they always check everything. My partner always wants to talk about books and politics and they all participate in that. But still, when things occur, they always check him to make sure he doesn’t have pressure sores and things like that, without even specifically mentioning it. So they do check sensibly. I have confidence in that.”*

Female partner of a patient with lung cancer

In addition to medical knowledge and skills, a number of interviewees mentioned proper communication about the diagnosis and prognosis as important for good care. However, the preferred communication styles differed between patients. Some patients appreciated the GP taking an ‘objective’ approach, presenting statistics and averages, as this was perceived to be useful for decision-making, while others did not want such information because they found this too much to face.

#### Theme 2: availability

When being asked about the role of their GP, interviewees often referred directly to the fact that he or she was available day and night and they could always reach the GP if the patient needed specific care or had urgent questions. Additionally, they often mentioned that it was not a problem for their GP to pay a home visit for extras such as injections or draining fluids, or stay a bit longer for a good chat. Some small critical remarks were made about the fact that patients first had to explain the whole situation to the GP’s practice assistants before they could reach the actual GP.

Some GPs were even willing to increase their availability further still for the cancer patient and their relatives by giving their private telephone number. Having a good (pre-existing) bond with the GP possibly influenced such additional service.

*“I have to say, I could call the GP 24 hours a day, and even had his private number. At a certain point he went on holiday for the weekend. That was when he said: ‘I can’t promise you that I will pick up the phone immediately but I will keep my eyes on it and will certainly call you back.’ That was really nice, you know.”*

Daughter of a patient with breast cancer

Similar experiences concerned the community nursing staff. Interviewees frequently mentioned that when a community staff member was called, they almost immediately responded by coming over. Some interviewees said that they never had the feeling that things were rushed, but indeed felt that the staff prioritized the patient’s well-being by spending a little bit more time with the patient.

*“My wife wanted a cigarette before she went to bed. Well, then the home-care staff sat down with her and then they would have a chat about all kinds of things, they gave her that opportunity. While they still had to put other people to bed, or wanted to go home or whatever. But still they had time, time for the patient, wonderful!”*

Male partner of patient with brain tumour

#### Theme 3: a focus on the person

Besides medical proficiency and availability, both patients and relatives stressed that they found it important that the professional is person-focused. The analysis revealed three aspects within this theme: showing personal interest, taking the patient and his or her relative seriously, and having a bond of trust. The first aspect, **personal interest from the professional,** was found to include attention and empathy, emotional support, affection, and the feeling that you could freely express your worries. If a GP is willing to get to know the patient as a person, he also tends to notice more details and can adapt the treatment accordingly. A relative stated that care should be on a personal level with attention for that specific patient’s illness in its unique situation.

*“Care should be highly personal, focused on the human being who is lying in the bed. Very personal. Not talking about general things such as statistics and averages* − *those kind of things. That’s all nonsense; you treat a specific patient, right?”*

Male patient with lung cancer

According to the interviewees, it is important that a professional is aware of the latest developments in the patient’s situation. This entails having either the GP or community staff regularly visiting the home or calling the patient (by appointment or unannounced) to check how the patient is doing and whether anything is needed, and to look at the current situation at home, while also considering the relatives.

*“Commitment. That is a GP, as I have now experienced, who feels that the moment something like this [cancer] occurs, they have the moral duty to contact you regularly. So by asking: ‘How are you? How is the treatment going? Are the medicines effective?’ It is her own interest in the patient that makes her say: ‘I want to be up to date on your health status.’ That is what I find important.”*

Male patient with lung cancer

Generally, this way of caring to stay up to date intensified as the final phase came closer. If community nurses dropped by regularly or between shifts, relatives said they felt calmer, knowing that someone would have a look at the patient’s situation. Moreover, showing interest in relatives and how they were coping with the situation, thereby preventing them from becoming overburdened, was appreciated by relatives, but also something that was missing in some cases. One relative argued that simply listening is highly valued.

*“If I have something that I can’t handle myself, I can always call them. That is so perfect. And you know they will listen. That they are loving and committed….You’re not a number, it comes straight from the heart.”*

Female partner of a patient with lung cancer

Secondly, **being taken seriously** is what relatives in particular stressed as important. Relatives want the best for the patient, although some experienced being pushed back as their concerns were not always taken seriously. For example, a daughter who recognized from her mother’s facial expressions that she was in pain, felt she was not being taken seriously as the GP initially believed that the daughter only wanted to increase the morphine dose. Furthermore, in some cases the GP only acknowledged the patient as their contact person and only wanted to discuss medical information with the patient.

Lastly, from the interviews it appears that having **good contact and a bond of trust** with the GP and community staff is perceived as important, as patients and relatives have to rely on them more and more as the illness progresses. Trust is highly valued in the relationship with caregivers. A bond of trust with medical professionals tends to grow during the illness trajectory. This can for example be a reason for preferring a GP to take over injections from unknown specialists. Besides, sharing a pre-existing (personal) bond of trust with care professionals can sometimes be a help as it can be a positive influence on caregivers’ willingness to be available or involved more.

#### Theme 4: proactivity

Patients and relatives considered a proactive attitude on the part of their GP and community nursing staff to be important. For example, interviewees stated that it relieved them of a huge burden if the professional had good organizational skills and **planned their care arrangements** with the medical specialist or other care professionals. Additionally, it was valued if a GP acknowledged the fact that his knowledge and proficiency regarding a specific type of cancer was limited, and referred the patient to another physician with more expertise.

*“She [the GP] had arranged everything already. ‘You can just go home and wait for a phone call from the hospital, because they will call you for an appointment.’ She says, ‘that will probably be tomorrow.’ And yes, on Friday I could already visit the lung specialist. Well, every time I have contact with her, or something has to be arranged or happens, she responds very alertly.”*

Male patient with lung cancer

Secondly, participants also expect their GPs to **provide them with important information in good time** about medical arrangements, discussions with other care providers or possibilities for community nursing. If patients were not being informed properly, it felt as if the care provider lacked interest in them. Also, relatives indicated a wish to be informed directly by the GP every now and then, as the patient might be forgetful.

Thirdly, professionals who take the initiative to **openly discuss the end of life and the associated future care**, were often seen as providing reassurance and clarity about future arrangements. Although end-of-life discussions could be confronting, patients said this made them more conscious of the finiteness of life and better able to anticipate future decisions. Generally, participants appreciated GPs speaking out at an early stage and naming concrete facts instead of avoiding difficult topics.

“*Yes, you can talk about everything; we discussed euthanasia, about no longer having treatment. And she [the GP] is also very open, like ‘you should arrange this like that, or you have to do it this way. Just bear in mind that if you can’t say something anymore then I also can’t help you anymore. Because you have to give approval yourself. So indicate it in good time.’ And then I think: that is what I like actually. The openness, directness in how she approaches you. And that they point out, ‘here I can help you and there I can’t.’ That’s when you know what you are up against and it also creates clarity for your partner.”*

Male patient with lung cancer

However, some relatives said that they would have expected more attention to be paid to how the end-of-life phase and dying process would affect them. They indicated that it is important for them to be included in such discussions as well, together with the patient. In general, community nursing staff were said to be open to discussing the end of life and end-of-life care, but often at the patient’s or family’s request or some other subtle indication. If needed, a palliative consultant or nurse experienced in cancer care was called to lead such conversations.

#### Theme 5: proper collaboration and information transfer between professionals

Patients and relatives found proper collaboration and transparency between healthcare professionals important. The collaboration between the GP and community nursing staff was generally experienced as positive. It was felt that they did not work in a vacuum but rather as partners. The ‘book’ (i.e. individual care plan) was often referred to as useful for the transfer of information. Care providers would open the book when arriving and write an account of what they had done before leaving. Moreover, it was mentioned that community staff and stand-ins were sufficiently up to date about the situation before arrival.

However, if multiple disciplines from primary and secondary care were involved in the care for a patient, a proper transfer of information was not always guaranteed. Some interviewees felt that GPs and specialist physicians worked separately and hardly communicated with each other, which could lead to errors and miscommunication. To avoid this, patient and relatives took a more active role themselves.

*“We learned to take control, otherwise you miss out on things. You see, he [the patient] has to deal with a nutritional team, with the oncologist in city A, the radiologist in city B, community nursing and the GP. Those are five channels. Well, that doesn’t align well. Because the GP should receive information, but the radiologist sends it to us and to the oncologist but not to my GP. And he [the GP] has no contact with the community nurses, because they are from another city, you understand? So if you don’t take good care of those things, and if they don’t arrange it, then that injection you should be getting monthly doesn’t happen, neither do medicines nor the blood sample that needs to be taken and analysed.”*

Female partner of a patient with cancer in the neuro endocrine system

#### Theme 6: clear and rapid procedures

A sixth theme that emerged during the analysis was the patients’ and relatives’ need for more flawless procedures and clear contact points. The fact that advanced cancer comes with all sorts of formalities caused numerous irritations among patients and relatives. It was frequently mentioned and emphasized that one had to be very alert or else you would not receive the care needed. Frustrations include bureaucratic matters in the healthcare sector, e.g. when needing medical-technical aids or reimbursements for the care received. Organizing the complex paperwork accurately and chasing up reimbursements costs energy and was seen as time-consuming; time that patients and relatives would rather have spent differently.

*“Borrowing things such as a wheel chair, commode chair, mattress, the things that were needed because she was at home, was difficult. A fuss, even though the palliative consultant was willing to help. But still, a statement from the GP was required and so it couldn’t go directly through the consultant. I got sick of it. These are the formalities, the right man has to order the right things in the right way with the right documents. You became so sick of it. And you do’nt want to have to bother about such things at that moment.”*

Male partner of a patient with a brain tumour

In addition, participants also stressed the importance of having clarity about one’s point of contact for help within the care trajectory. The GP was frequently mentioned as the central person and source of information. To others, nursing staff who were experienced with cancer were particularly useful. However, some patients reported a lack of coordination, and thus vagueness about one’s central contact, which caused them a lot of distress, e.g. in instances when they needed a repeat prescription for their medicine and they did not know who to turn to. They felt they were being sent back and forth between the primary and secondary care providers, e.g. in instances where the GP had limited expertise, or where multiple disciplines were involved due to metastases or comorbidities*.*

### Interrelatedness of themes

**Fig. 1:**
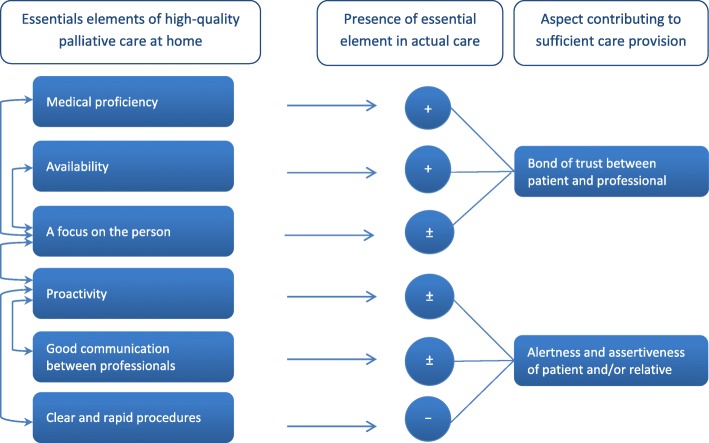
A schematic overview of the interrelatedness of essential elements of high-quality palliative care at home. (+) Element generally present in the care participants received. (±) Element sometimes (not) present in the care participants received. (−) Element generally not present in the care participants received

Figure 1 presents a schematic diagram of the main themes and how they interrelate. The arrows on the left show which themes can have a positive or negative effect on other themes if (not) being successfully met in the provision of care. The figure also shows whether these essential elements were generally present in the care participants received, and the factors that contributed to this.

Interrelatedness can for instance be seen between the themes of ‘availability’ and ‘a focus on the person’, e.g. in cases where a professional is more willing to check on the patient or provide his private phone number, when he or she and the patient share a good relationship and a mutual bond of trust. Vice versa, if a professional is very person-focused, people are likely to feel they can call this professional whenever needed. The foundation for a good bond is already laid with a proper diagnosing procedure. The interviews showed that trust in a professional’s medical proficiency is an underpinning factor for further GP care in the illness trajectory. The interviews also showed that a proactive attitude of professionals tends to have a positive influence on collaboration with other professionals. If a professional showed initiative and actively participated in managing an optimal illness process, patients seemed more satisfied with the information that was provided to them, and seemed more confident in finding their ways through the maze of bureaucratic procedures.

However, depending somewhat on the extent to which professionals were proactive, patients and relatives frequently reported that they also needed to be alert and keep sharp watch on the information transfer between professionals and the formal care arrangements themselves, in order to avoid misunderstandings and errors.

*“You have to watch things closely yourself. Yes, it is simply necessary in the Netherlands. Because else you will miss out on things, or it won’t work out the way you want it to. And that is a pity. But I wouldn’t know how to change it, since you have to deal with various specialists and multiple disciplines. But someone who isn’t alert, could possibly miss out on care that he does deserve.”*

Daughter of a patient with ovarian cancer

To make sure they did not miss out on proper care, the interviewees often took a more active role. For example, if they were insufficiently informed or had a fairly passive GP, they searched and asked for answers or additional support themselves. Frequently, they had to dive into matters themselves and update the professionals. An alert and assertive attitude on the part of patients and relatives turned out to be a factor which could contribute to sufficient care provision in three themes in particular. It could enhance a proactive attitude of professionals, facilitate smooth collaboration between care providers, and improve the management of formal procedures (see Fig. 1). Participants expressed worries about other patients who might be less vigilant regarding the care they receive, or who have no family to support them in this.

## Discussion

The aim of this study was to get insight in the elements that patients with advanced cancer and relatives consider essential for high-quality palliative care, and whether these essentials are present in the actual care they receive. The subjective views of patients and relatives repeatedly show six elements that are considered essential for good-quality primary palliative care provision: medical proficiency, availability, a focus on the person, proactivity, proper collaboration and information transfer between professionals, and clear and rapid procedures. These six essential elements are interrelated and can be supported by a mutual bond of trust between the care professional and the patient, and by an alert and assertive attitude on the part of the patient and his family. The interviews also reveal that most interviewees had positive care experiences with regard to the aspects they consider essential for good-quality primary palliative care. However, the requirements of ‘proper information transfer between professionals’ and ‘clear and rapid procedures’ were mentioned as more difficult to meet in actual practice.

Our findings are in line with those from previous research. For example. They are closely related to the aspects that Steinhauser et al. (2000) identified in a quantitative study among 1462 patients, bereaved family members and care providers as to what they considered important at the end of life [15]. Among the highest ranked items were: to be kept clean, have a nurse with whom one feels comfortable, know what to expect about one’s physical condition, have someone who will listen, have a physician with whom one can discuss fears and knows one as a whole person, and know that one’s physician is comfortable talking about death and dying. These are items that are closely related to the themes found in this study of ‘medical proficiency’, ‘availability’, ‘a focus on the person’ and ‘proactivity on the part of the professional’. In a meta-ethnographic study of 19 qualitative studies exploring the experiences of (mainly) relatives of advanced cancer patients with palliative home-care services, the themes of ‘availability’ and ‘medical proficiency’ were also identified as two overarching components of home palliative care, contributing to meet the participants’ core need for security [16]. Studies that explored the perspective of GPs on good end-of life care revealed that GPs also perceive (extensive) availability, personal interest, medical proficiency and collaboration between care providers, vital for good-quality palliative care [17, 18]. Moreover, having intensive contact with the patient is acknowledged by GPs as an underpinning factor for avoiding hospitalizations as this enables better monitoring and guidance of the patient and his family in a holistic way through the illness trajectory [19]. The shared priorities of what GPs and patients regard as important for primary palliative care show a common ground for the sound provision of care.

Whereas our findings indeed confirm that having trust in professional proficiency seems key to the overall care experience (as not trusting the medical proficiency of a care professional may have led to the ending of the care relationship earlier in the illness trajectory), our study adds to this by pointing towards other essential elements as well, namely ‘proper collaboration and information transfer between care professionals’ and ‘clarity of procedures’. In our study, participants reported their worries and frustrations regarding miscommunication between the GP and the medical specialist, and time-consuming procedures and formalities. Besides being alert and assertive themselves, interviewees considered proactive professionals to be essential in improving collaboration between care professionals and managing bureaucratic issues. These findings are in line with and elaborate on the results of the quantitative study by Heins et al. (2018), which showed that more attention could be given to the provision of adequate information and to the provision of support when fatigue is an issue, i.e. when the patient is less able to be alert and assertive [10]. Studies investigating the GP perspective found that GPs themselves also complain about slow or non-existing information sharing between providers, as well as insufficient care coordination [20, 21]. Some proactively try to overcome these obstacles through direct contact with patients and physicians, and by building networks of trusted care providers. Proactivity on the part of GPs has been identified as one of three conditions for enabling end-of-life discussions [21], in addition to the specialist being realistic to patients about the limits of what treatment can achieve, and the specialist informing the GP properly. Earlier and better inter-professional communication between specialists and GPs seems required, and could for instance be facilitated by the proper electronic transfer of patient information [22, 23] and guidelines on e.g. key moments when communication is required and maximum periods during which information transfer should take place. Better inter-professional communication and coordination, but also less bureaucracy and clearer procedures for obtaining resources and services, will reduce the need for patients and relatives to be vigilant in order to receive good care. In particular, patients who are less assertive themselves, have no family to support them or have limited health literacy will benefit from this.

Finally, although the WHO states that palliative care also includes support for the family [24], the interviews revealed that some relatives felt excluded or not taken seriously in the care process or end-of-life discussions. This is in line with previous findings showing that relatives felt that good support is given to the patient, while they themselves are in need of more psychosocial support and personal attention [25, 26]. Sufficient attention for relatives is of great importance, as they are important partners in the patient’s care; if a relative is distressed, they may not be providing their sick loved one with emotional support or may not really be there for their loved one. It is therefore important to fully include relatives in the patient-physician encounters and to prevent them from becoming overburdened [27, 28].

### Methodological considerations

A strength of this study is that the subjective views of patients and relatives are combined, giving an in-depth and comprehensive picture of what they consider essential in palliative care. By reflecting on the care they received themselves, findings reflect actual care practice and avoided answers that were socially desirable or rather abstract. The essentials we identified are applicable to the care provided both by GPs and by community nurses, although the examples that were given mainly concerned the practice and role of GPs as not all patients received community nursing care. This is caused by our recruitment strategy in which we made use of the framework of a quantitative study in which GPs identified eligible patients. GPs might have selected patients with whom they had a good relationship, possibly resulting in too positive a picture of the patient-GP interaction. Furthermore, the participants were relatively highly educated, which could have led to a bias in the findings, as well as might limit their generalizability. However, the fact that even these people, who were relatively highly educated and have a good relationship with their GP, have difficulties with bureaucracy and formalities underlines the importance of simplifying this.

## Conclusion

Medical proficiency, availability, a focus on the person, proper information transfer between care professionals, clear and rapid procedures and proactivity on the part of GPs and community nursing staff are considered essential for good palliative primary care at home. Although these essential elements were often fulfilled in the actual experience of patients and relatives, improvements are warranted in particular with regard to collaboration and information transfer between professionals, and current bureaucratic procedures. Particularly when patients and relatives are less assertive, it is important for care professionals to ensure that the identified essential elements for high-quality palliative care at home are met.

## Data Availability

Meta-data of analysed transcripts are available from the corresponding author on reasonable request.

## Abbreviations

GP: General Practitioner
WHO: World Health Organization
